# Idaho Springs

**Published:** 1874-03

**Authors:** 


### IDAHO SPRINGS.

SITUATED on South Clear Creek, thirty-five miles by stage from Denver City.

The temperature of the water is about 100° Fahrenheit, and the flow sufficient to supply two large swimming baths with a renewed charge of pure water every twenty-four hours.

ANALYSIS OF ONE GALLON.

| | |
|---|---|
| Carbonate of Soda | 30.80 |
| Carbonate of Lime, | 9.52 |
| Carbonate of Magnesia | 2.88 |
| Carbonate of Iron | 4.12 |
| Sulphate of Soda | 29.36 |
| Sulphate of Magnesia | 18.72 |
| Sulphate of Lime | 3.44 |
| Chloride of Sodium | 4.16 |
| Chlorides of Calcium and Magnesia, of each a trace. | |
| Silicate of Soda | 4.08 |
| Grains | 107.00 |

The water yields a small proportion of carbonic acid gas only. The medicinal characteristics of the spring are antacid, alterative, and in many cases slightly laxative. Its external use as a bath will be found beneficial in cases of rheumatism and diseases of the skin.

The temperature is sufficiently high in winter to make the bath pleasantly warm, and can be regulated to suit all seasons and all classes of bathers. The Ocean Bath House is open at all seasons, and has a swimming bath twenty-four by forty feet, and four feet in depth, the water in which is renewed every twenty-four hours from the spring; private baths for ladies, with female attendants; private baths for gentlemen, and hot and cold shower baths, all in first class order, and dressing rooms comfortable at all seasons. Besides the springs above described there has been discovered near Soda creek a SODA SPRING, whose waters are nearly as sparkling and effervescent as those charged with carbonic acid gas for use at soda fountains. At this watering place tourists will find first class hotel accommodations, good liveries, and delightful drives over fine roads, surrounded by unrivalled scenery.

